# Supplementary material for: Euglena gracilis Enhances Innate and Adaptive Immunity through Specific Expression of Dectin-1 in CP-Induced Immunosuppressed Mice
Source: Nutrients. 2024 Sep 18;16(18):3158. doi: 10.3390/nu16183158 (PMC11434765; doi:10.3390/nu16183158)
Supplement: Supplementary file 1 [file nutrients-16-03158-s001.zip › nutrients-3185581-supplementary.pptx]

## Slide 1
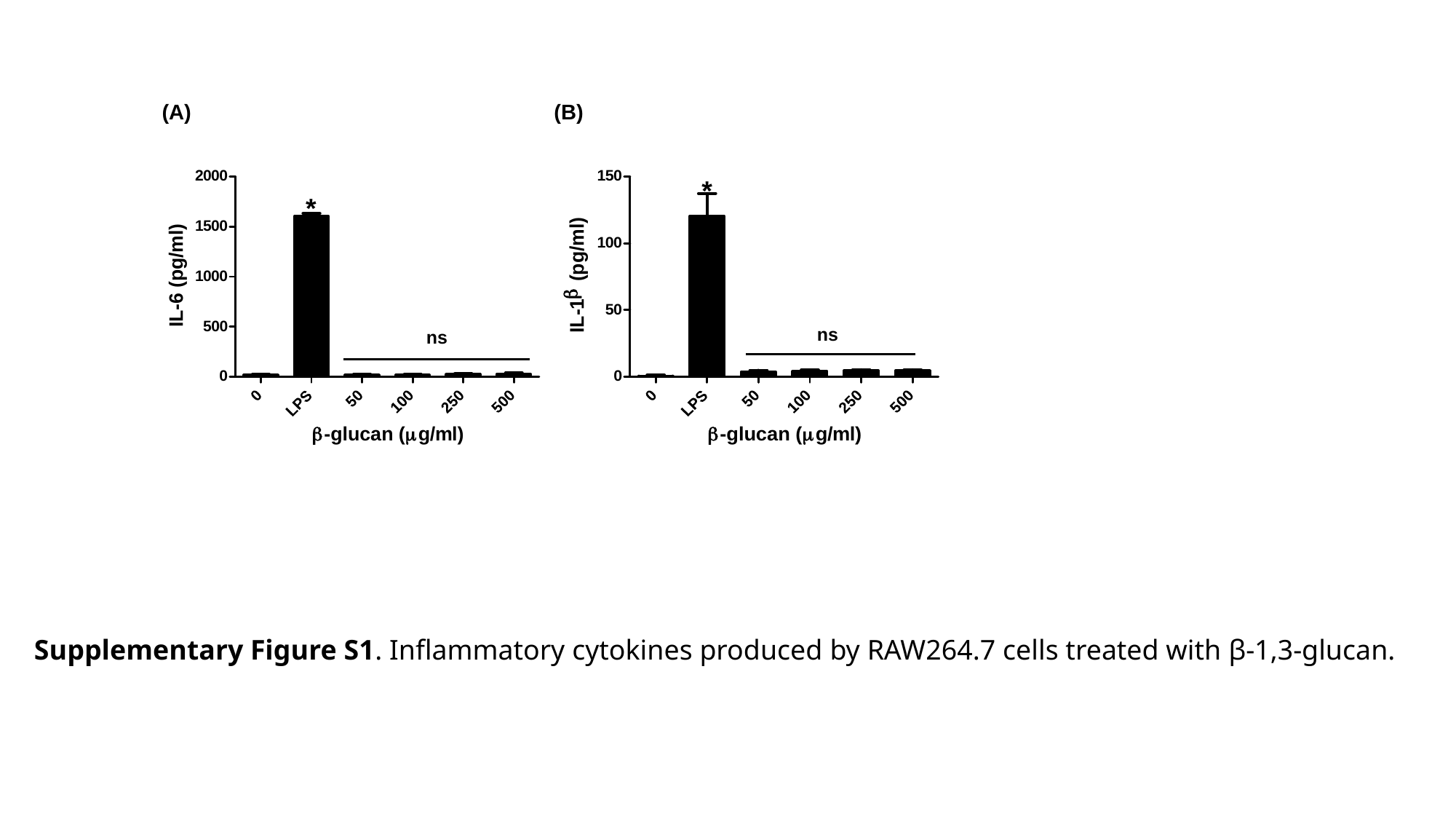

(A)
(B)
*
ns
*
ns
Supplementary Figure S1. Inflammatory cytokines produced by RAW264.7 cells treated with β-1,3-glucan.

## Slide 2
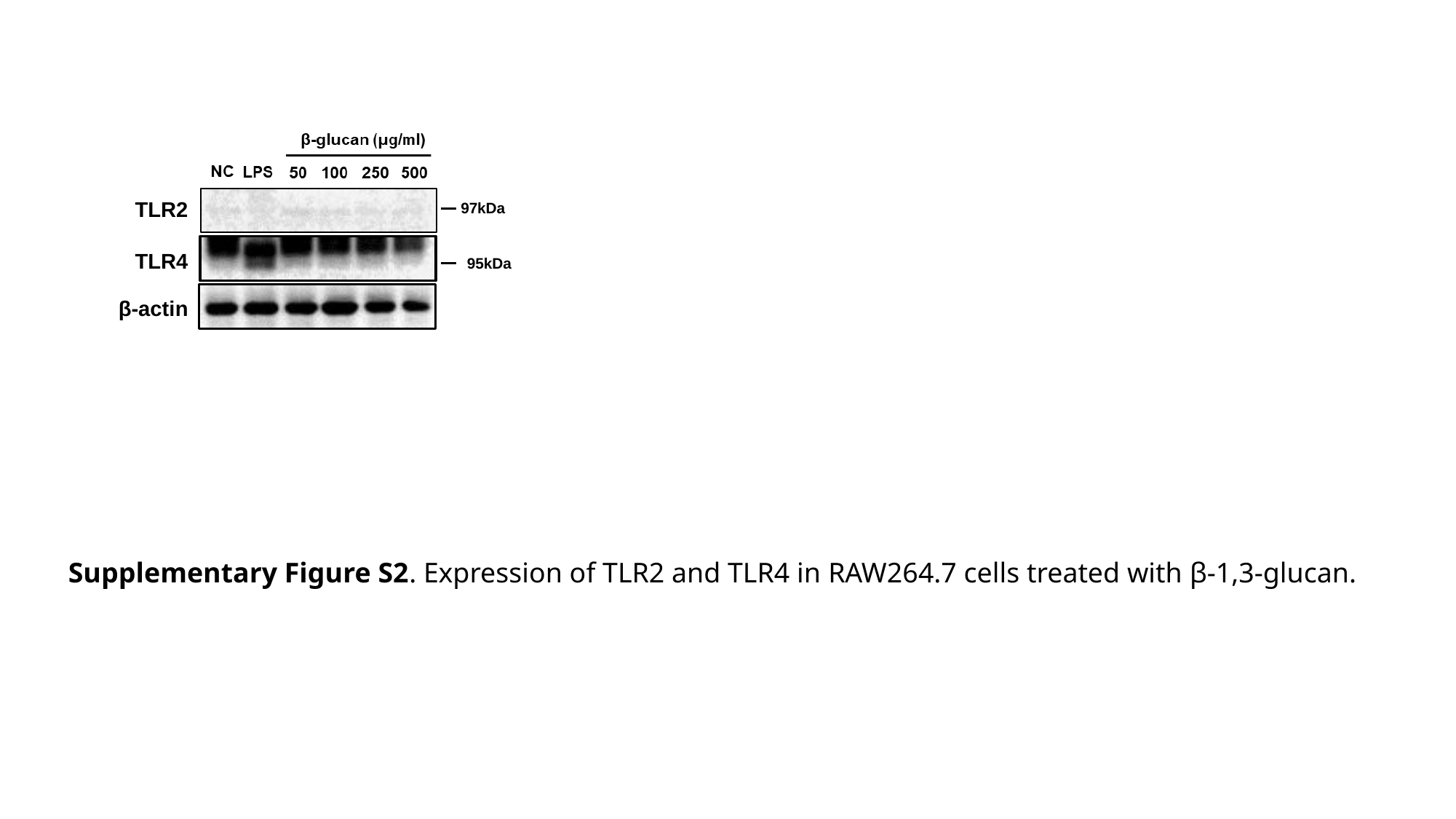

TLR2
97kDa
TLR4
95kDa
β-actin
Supplementary Figure S2. Expression of TLR2 and TLR4 in RAW264.7 cells treated with β-1,3-glucan.

## Slide 3
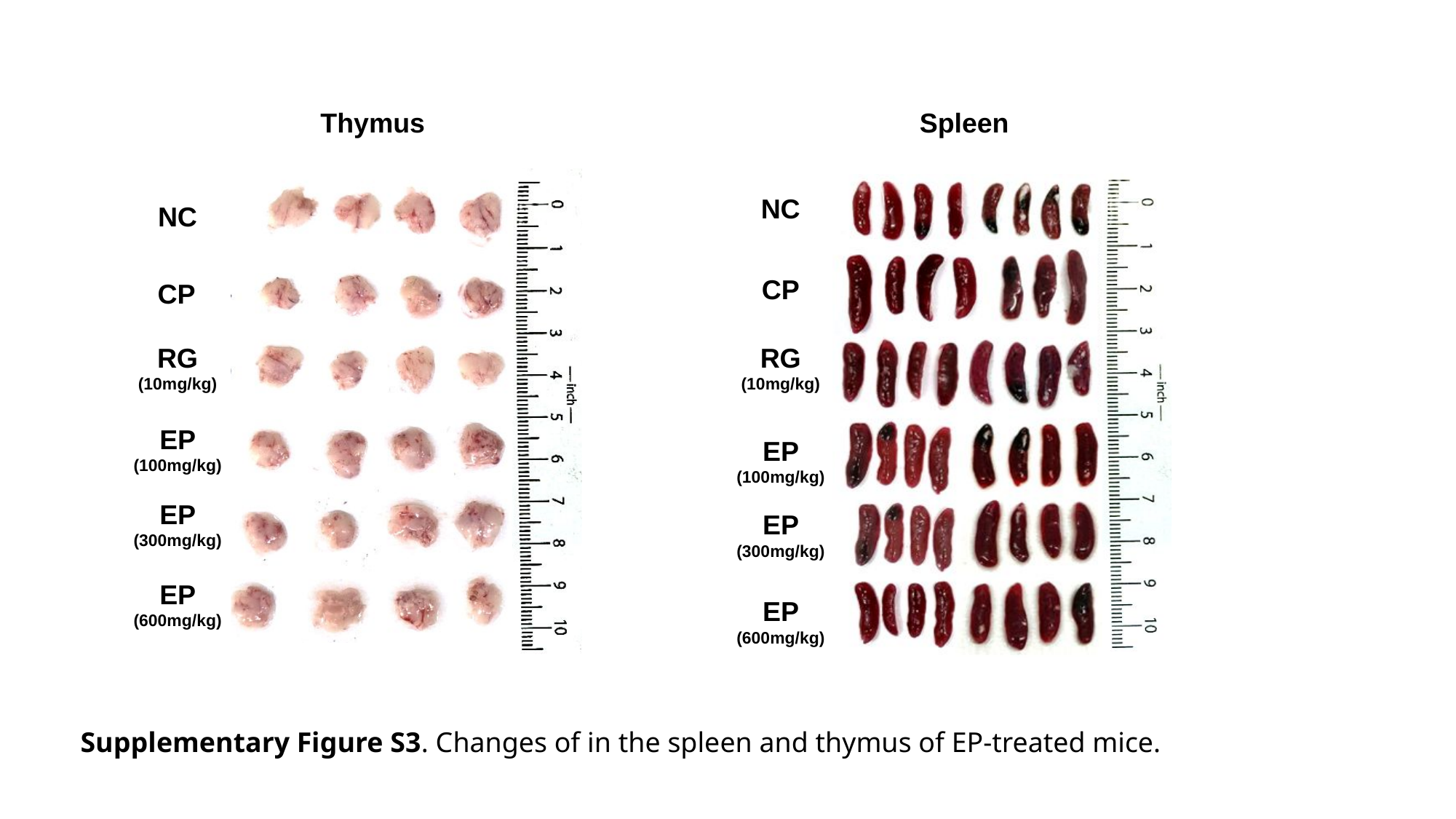

Thymus
Spleen
NC
CP
RG
(10mg/kg)
EP
(100mg/kg)
EP
(300mg/kg)
EP
(600mg/kg)
NC
CP
RG
(10mg/kg)
EP
(100mg/kg)
EP
(300mg/kg)
EP
(600mg/kg)
Supplementary Figure S3. Changes of in the spleen and thymus of EP-treated mice.
